# Supplementary material for: Blood and lymphatic systems are segregated by the FLCN tumor suppressor
Source: Nat Commun. 2020 Dec 9;11:6314. doi: 10.1038/s41467-020-20156-6 (PMC7725783; doi:10.1038/s41467-020-20156-6)
Supplement: Supplementary file 1 — Supplementary Information [file 41467_2020_20156_MOESM1_ESM.pdf]

Supplementary Information

Blood and lymphatic systems are segregated by the FLCN tumor suppressor

Ikue Tai-Nagara et al.

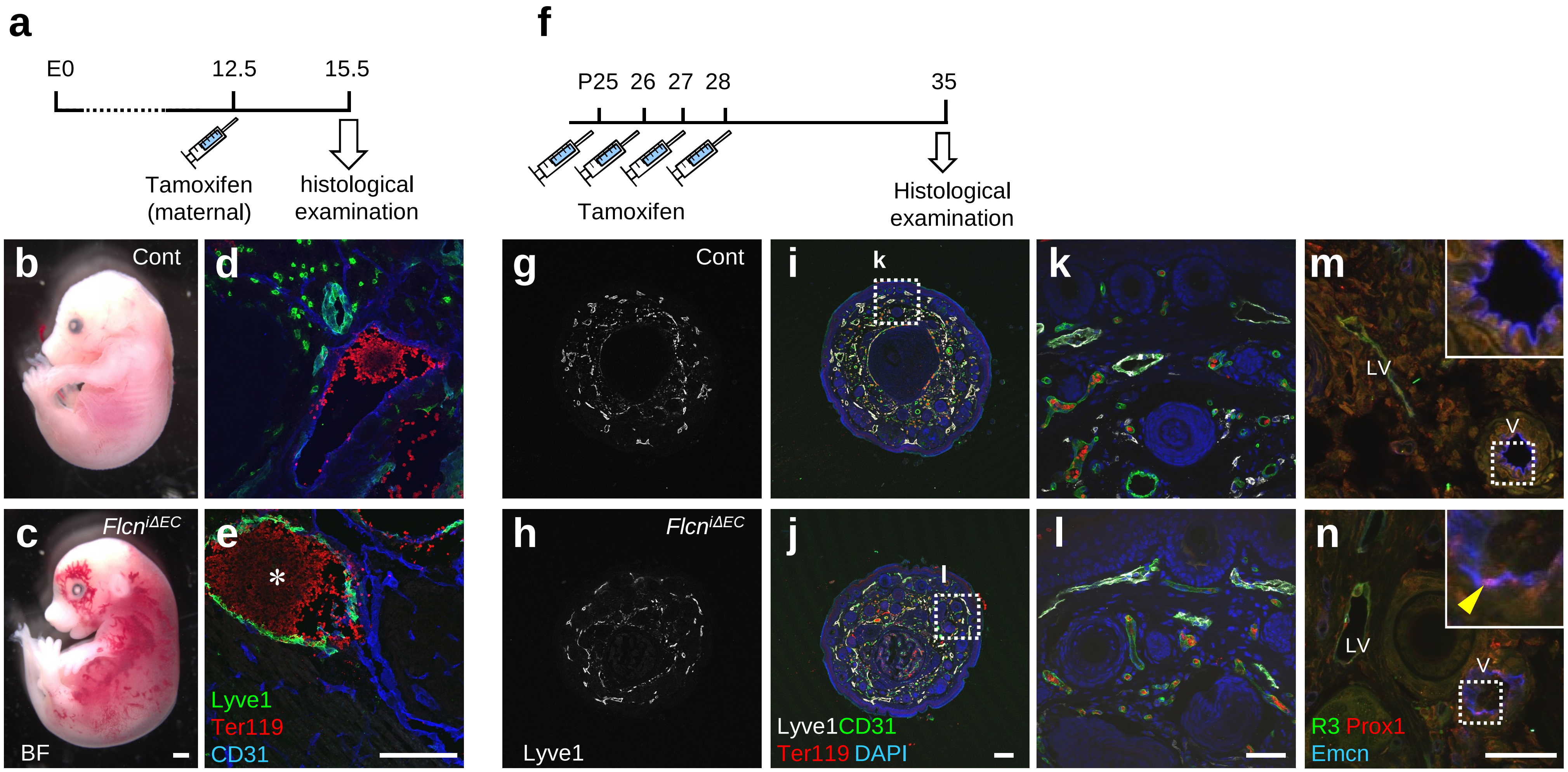

Supplementary Figure 1

Supplementary Figure 1. Flcn deficiency causes blood-filled and dilated lymphatics during embryogenesis.

(**a**) Protocol for tamoxifen injection into pregnant females. (**b, c**) Images of control and $\mathit{Flcn}^{i\Delta EC}$ mice at embryonic day E15.5. (**d, e**) Immunohistochemical analysis of coronal sections of E15.5 embryos. Flcn^{iEC} embryos show enlarged and blood-filled lymphatic vessels (asterisks). (**f**) Protocol for tamoxifen injection. (**g–n**) Immunohistochemical analysis of tail sections at P35. $\mathit{Flcn}^{i\Delta EC}$ mice show ectopic Prox1 expression in veins (V) (arrowheads) but not blood filling in lymphatic vessels (LV). Scale bars: 1 mm (**b, c**); 200 µm (**d, e, g–j**); 50 µm (**k–n**).

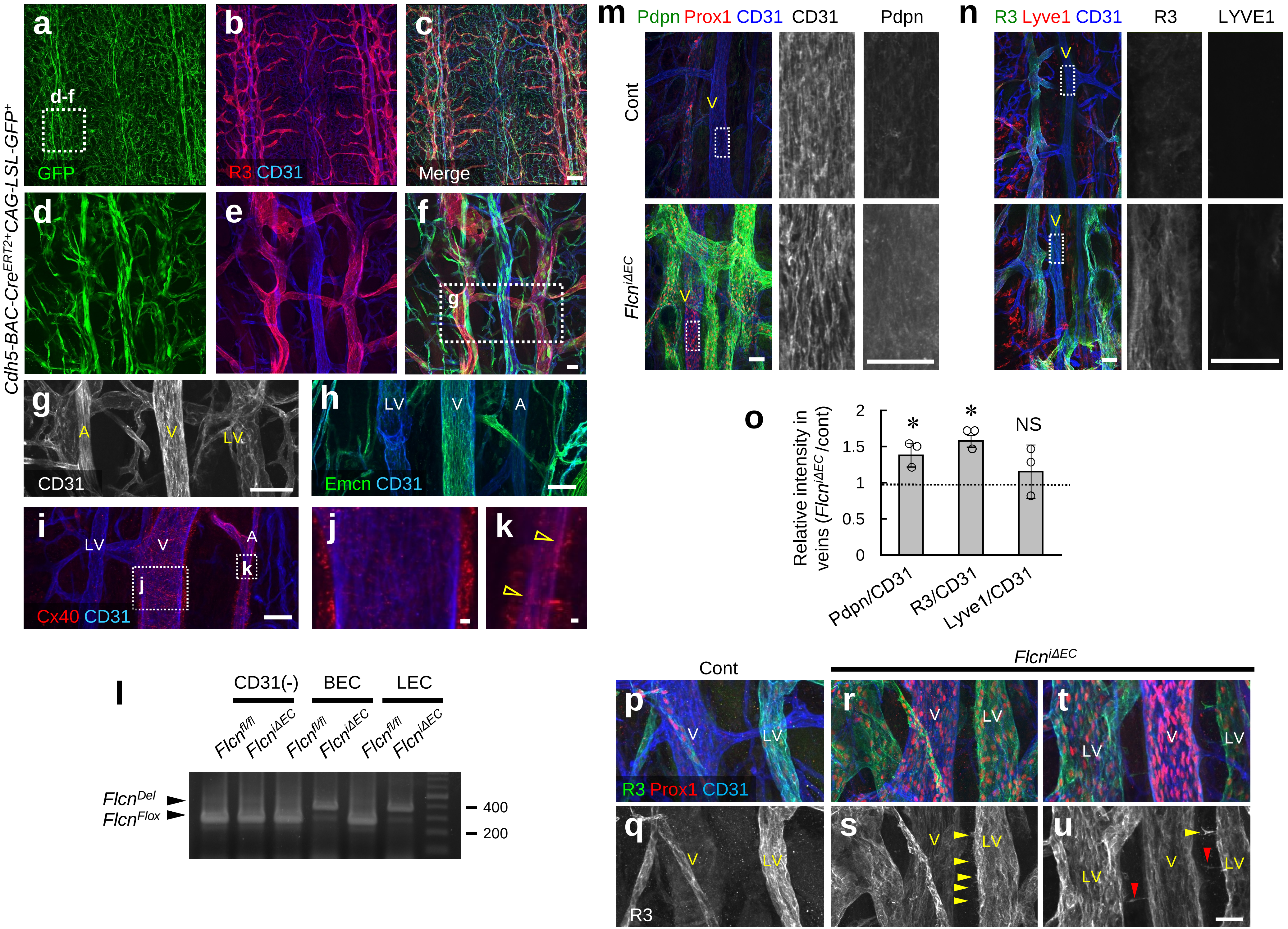

Supplementary Figure 2

Supplementary Figure 2. Characterization of LEC-biased veins in Flcn$^{i\Delta EC}$ mice.

(**a–k**) Immunohistochemical analysis of tail whole-mounts at P8. Arteries (A), veins (V), and lymphatic vessels (LV) are readily discriminated according to the shape of endothelial cells or expression of Emcn and Cx40. Cx40 is also expressed by vascular mural cells (**k**). Open arrowheads indicate Cx40 expression in arterial endothelial cells. (**l**) PCR analysis of genomic samples from P8 mesentery separated using Dynabeads. BEC, blood endothelial cells; LEC, lymphatic endothelial cells. (**m–o**) Immunohistochemical analysis/quantification of tail whole-mounts at P8 (n = 3) showing the differential intensity in Pdpn and R3. V, veins. (**p–u**) Immunohistochemical analysis of tail whole-mounts at P8. Abnormal sprouting from lymphatic vessels (LV) toward veins (V) (yellow arrowheads) and bridging of filopodia between veins and lymphatic vessels (red arrowheads) are detected in *Flcn*$^{i\Delta EC}$ mice. Scale bars: 200 µm (**a–c**); 50 µm (**d–i, m, n; p–u**); 5 µm (**j, k**). The comparisons between the averages of the two groups were evaluated using the two-sided Student's t-test. $^{*}P$ <0.05; NS, not significant. Data are presented as the mean ± SD. Source data are provided as a Source Data file. Unprocessed original scans of blots are shown in Source Data file.

a

	Cont	$Flcn^{i\Delta EC}$
Estimated Number of Cells	6,814	4,459
Mean Reads per Cell	51,605	80,612
Median Genes per Cell	2,085	2,273
Valid Barcodes	98.3 %	98.3 %
Sequencing Saturation	60.5 %	66.0 %
Q30 Bases in Barcode	97.8 %	98.0 %
Q30 Bases in RNA Read	67.3 %	66.6 %
Q30 Bases in UMI	97.7 %	97.8 %
Reads Mapped Confidently to Transcriptome	52.6 %	56.1 %
Reads Mapped Confidently to Exonic Regions	55.7 %	59.2 %
Reads Mapped Confidently to Intronic Regions	23.9 %	21.6 %
Reads Mapped Confidently to Intergenic Regions	6.0 %	5.4 %

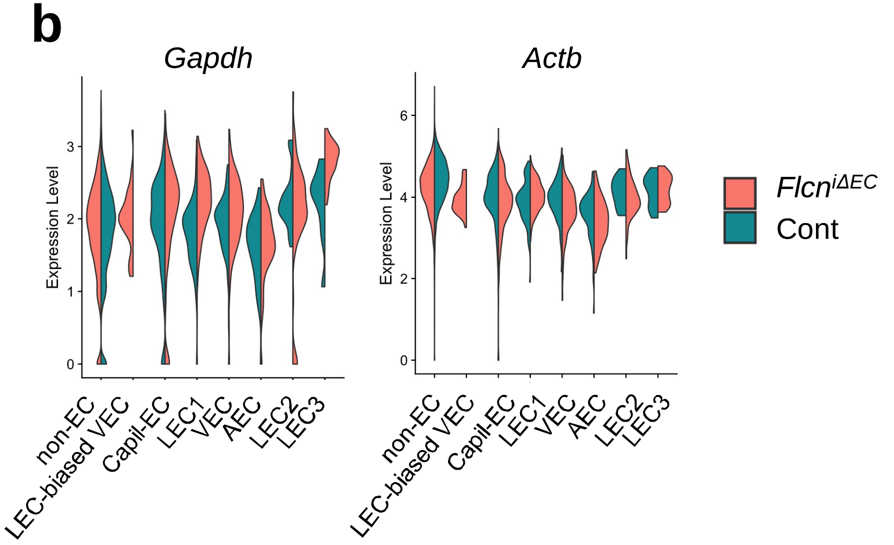

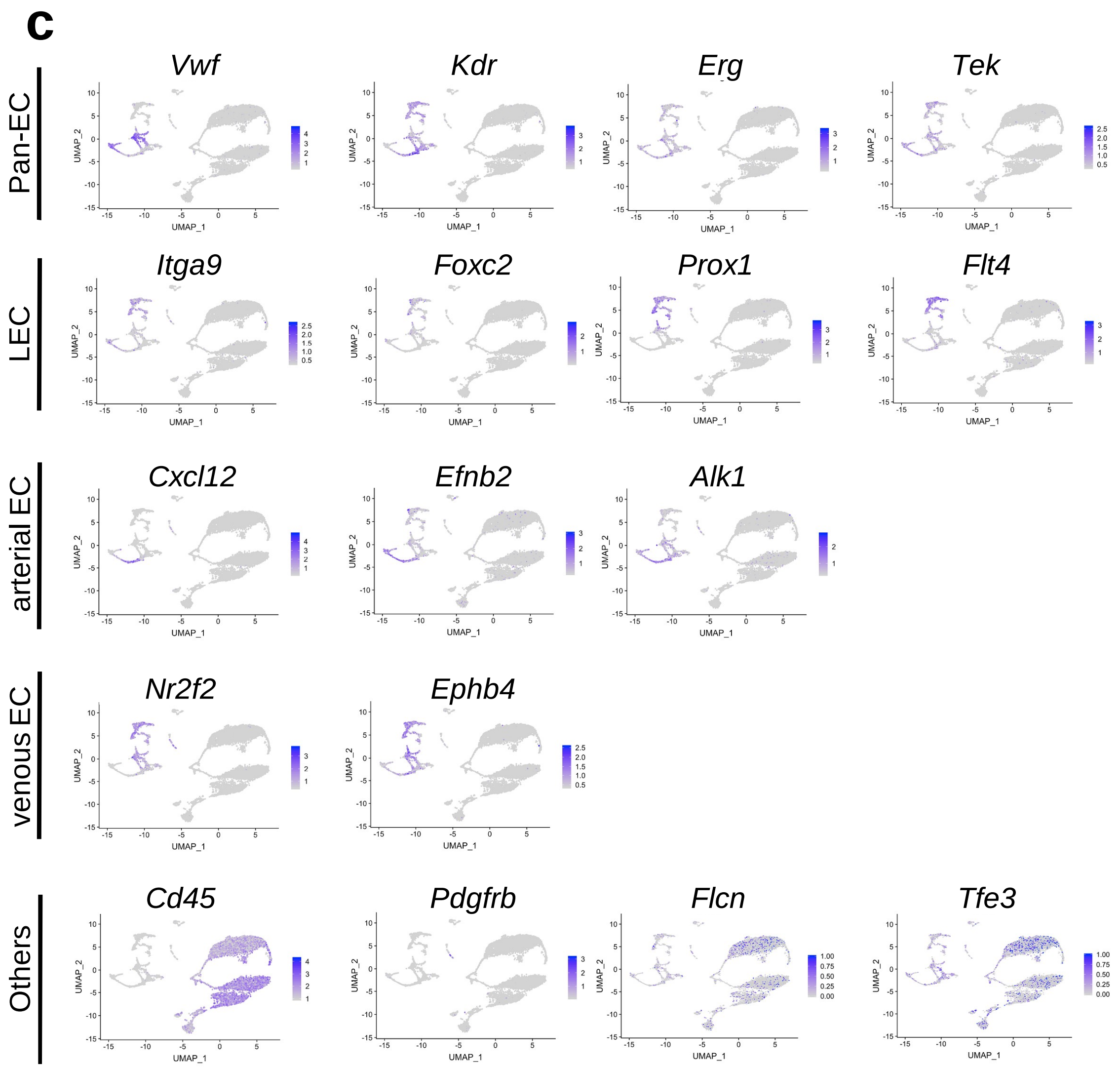

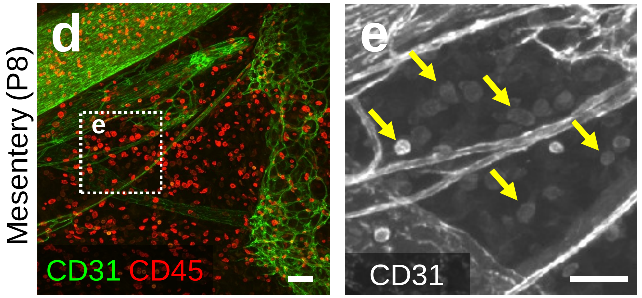

f

Relative gene expression

	pct			AEC/VEC		AEC/LEC1		VEC/LEC1	
	AEC	VEC	LEC1	p_val	avg_log FC	p_val	avg_log FC	p_val	avg_log FC
Flcn	0.131	0.262	0.245	0.00061	-0.0447	0.000973	-0.05615	0.940	-0.0115
Tfe3	0.345	0.497	0.312	0.00087	-0.063	0.559	0.015	4.58E-06	0.078

Supplementary Figure 3

Supplementary Figure 3. Quality control and characterization of endothelial clusters in scRNA-seq.

(**a**) Statistics of cells analyzed in the scRNA-seq. (**b**) Violin plots for internal control genes for the comparison of control and *Flcn*$^{i\Delta EC}$ cells. (**c**) UMAP plots of CD31^{+} cells from the mesentery of P8 mice showing expression of marker genes with enriched expression for each cluster. (**d, e**) Immunohistochemical analysis of a mesenteric whole-mount sample at P8. CD45^{+} hematopoietic cells show weak expression of CD31 (arrows). (**f**) Relative expressions of *Flcn* and *Tfe3* and their comparison among AEC, LEC, and LEC1 clusters. The data are automatically extracted from scRNA-seq using Seurat. Statistics of cells analyzed in the scRNA-seq. Scale bar: 50 µm.

a

Top 10 genes upregulated in LEC-biased VEC (vs VEC)

	p_val	avg_logFC
H19	8.41E-30	2.5530531
Olfm1	6.69E-19	1.77473616
Odc1	5.30E-17	1.60892629
S100a6	5.64E-19	1.40328051
Fxyd6	3.43E-19	1.39590312
Igf2	1.55E-21	1.38177327
Cldn11	2.52E-18	1.36500595
Plat	4.70E-14	1.36159801
Neo1	3.46E-19	1.30035665
Dusp2	4.32E-16	1.25007994

Top 10 genes downregulated in LEC-biased VEC (vs VEC)

	p_val	avg_logFC
Ctla2a	3.18E-18	-2.3520713
Vwf	7.92E-22	-2.1293523
Fabp4	1.45E-16	-2.031616
Mgp	1.08E-15	-1.904049
Sparc	1.09E-20	-1.8302322
Eln	1.35E-16	-1.6955331
Fbln5	2.26E-19	-1.6124229
Ehd4	4.32E-20	-1.5235756
Anxa3	4.08E-21	-1.5226017
Nrp1	5.48E-19	-1.4839603

Top 10 genes upregulated in LEC-biased VEC (vs LEC1)

	p_val	avg_logFC
Meg3	2.01E-64	2.10663918
Mgp	1.87E-25	1.70246878
Eln	1.44E-08	1.61712873
Slc6a6	1.87E-18	1.26254748
Id2	1.22E-19	1.23072009
Edn1	2.81E-15	1.21106757
Aqp1	1.28E-26	1.18882596
Stmn1	5.26E-19	1.11479759
Flt1	2.99E-26	1.09466916
Mdk	7.35E-08	1.07293742

Top 10 genes downregulated in LEC-biased VEC (vs LEC1)

	p_val	avg_logFC
Ccl21a	8.15E-11	-3.8679798
Mmrn1	4.48E-23	-2.3300044
Fgl2	4.66E-16	-1.9744931
Nts	3.26E-11	-1.9255911
Lyve1	1.48E-06	-1.9169692
Gpihbp1	5.61E-09	-1.5003541
Pard6g	3.81E-22	-1.4670652
Lrrn4cl	1.36E-10	-1.4169248
Aebp1	3.65E-13	-1.2666014
Lpl	1.54E-13	-1.2537202

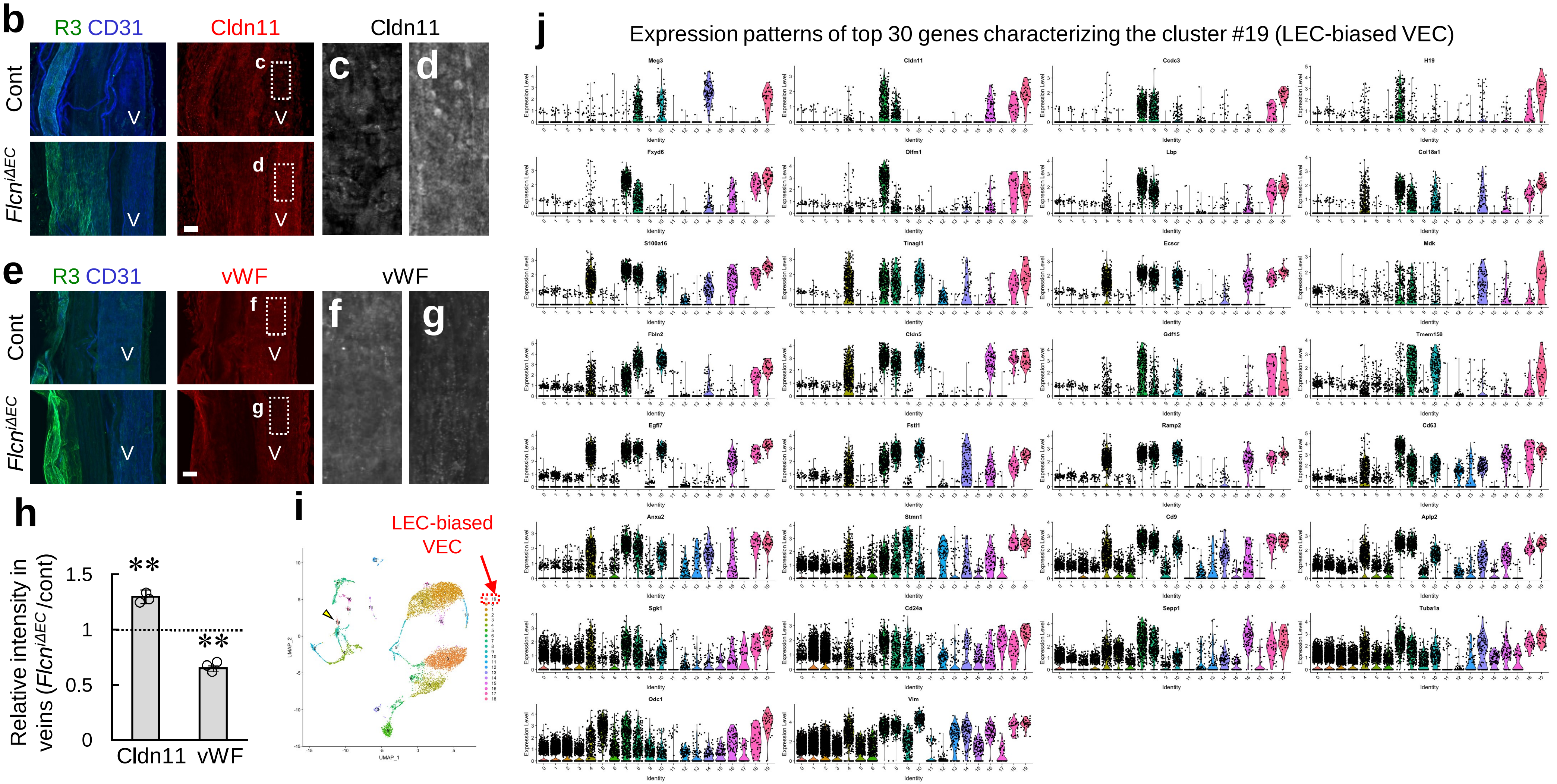

Supplementary Figure 4

Supplementary Figure 4. Molecular profile of LEC-biased venous endothelial cells.

(**a**) Lists of the top ten up- or down-regulated genes in LEC-biased VEC *versus* VEC, and in LEC-biased VEC *versus* LEC1. P-value and average log 2 fold change are shown. Full lists of genes are appended to **Supplementary Tables 1 and 2**. (**b–d**) Immunohistochemical analysis/quantification of mesenteric whole-mounts at P8 (n = 3) showing the differential intensity of Cldn11 and vWF; V, veins. (**e, f**) Violin plots of the top 30 genes characterizing the cluster of Prox1$^+$ VECs. All of the genes characterizing the cluster of LEC-biased VECs are strongly expressed by both VECs and LECs. Scale bars: 50 µm. The comparisons between the averages of the two groups were evaluated using the two-sided Student's t-test. ***P* <0.01. Data are presented as the mean ± SD. Source data are provided as a Source Data file.

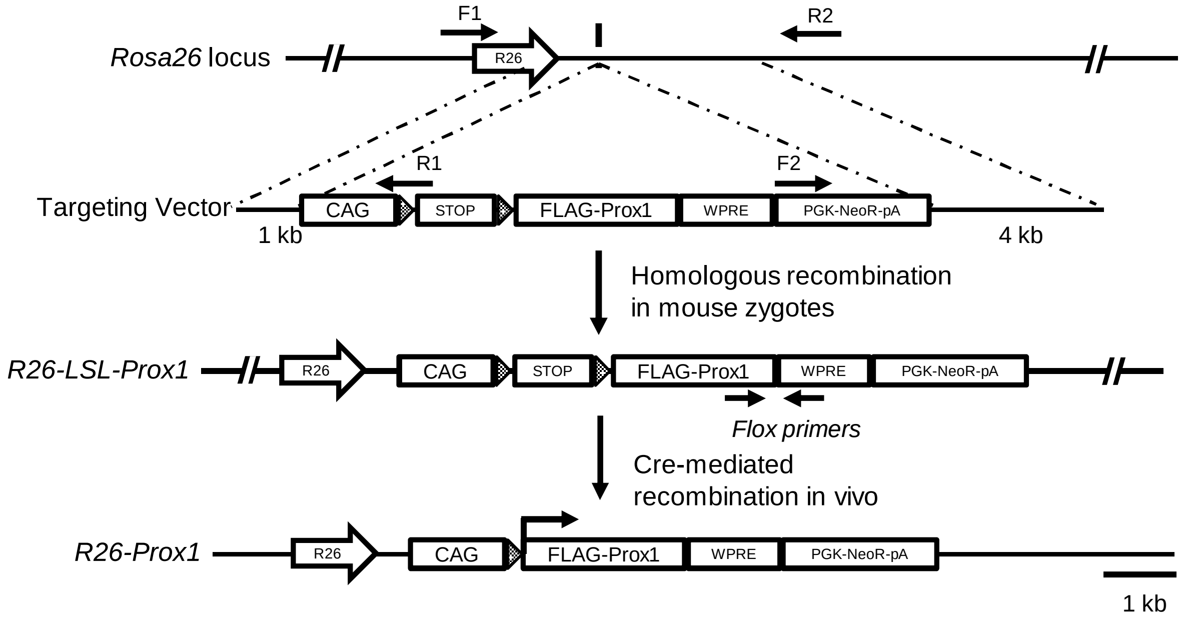

Supplementary Figure 5

Supplementary Figure 5. Generation of R26-LSL-Prox1 mice

Schematic diagram depicting the construct used for ROSA26-CAG-lox-STOP-lox-Prox1 (*R26-LSL-Prox1*) mice. A CAG-LoxP-Stop-LoxP-Prox1 construct was targeted to the Rosa26 locus in mouse zygotes via homologous recombination through CRISPR-Cas9-mediated genomic targeting. Cre-mediated recombination of CAG-LoxP-Stop-LoxP-Prox1 deletes the “floxed” stop cassette to generate CAG-Prox1.

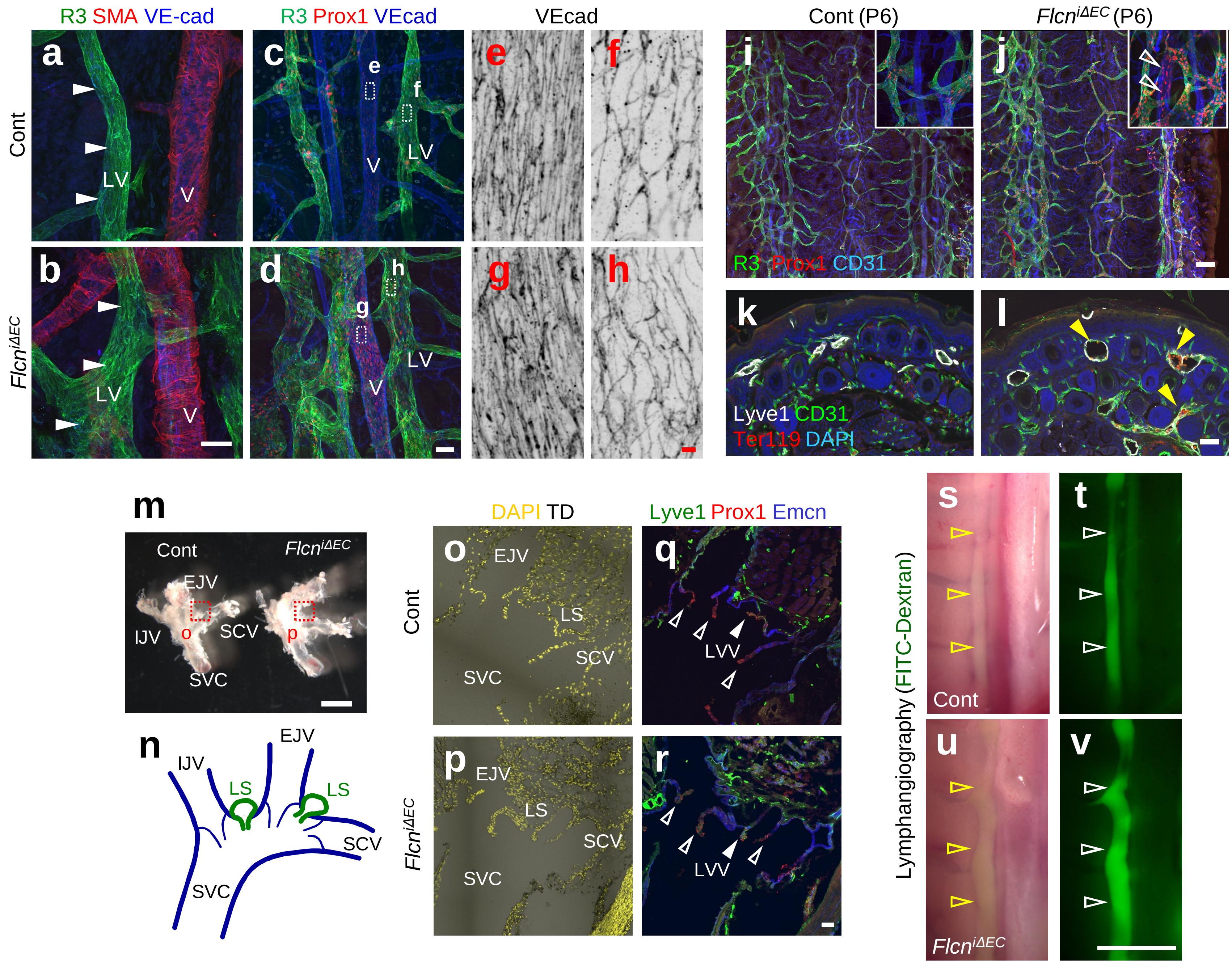

Supplementary Figure 6

Supplementary Figure 6. Detailed characterization of phenotypes in Flcn$^{i\Delta EC}$ mice.

(**a–h**) Immunohistochemical analysis of tail whole-mounts at P8. Adherens junctions of veins (V) and lymphatic vessels (LV) are intact in *Flcn*$^{i\Delta EC}$ mice (**e–h**). LVs are not ectopically covered with smooth muscle cells (closed arrowheads in **a, b**) in *Flcn*$^{i\Delta EC}$ mice. (**i–l**) Immunohistochemical analysis of tail whole-mounts (**i, j**) or sections (**k, l**) at P6. Venous Prox1 expression (open arrowheads) and blood-filled lymphatics (closed arrowheads) are detected in *Flcn*$^{i\Delta EC}$ mice without subcutaneous hemorrhage. (**m, n**) Bright filed view and schematic diagram depicting the left venous angles. LS, lymph sacs; EJV, external jugular veins; IJV, internal jugular veins; SCV, subclavian veins. (**o–r**) Section immunohistochemistry of left venous angle at P8. No apparent abnormality is detected in lymphovenous valves (LVV; closed arrowheads) in *Flcn*$^{i\Delta EC}$ mice. Open arrowheads, venous valves. (**s–v**) Bright field (**s, u**) and fluorescent microscopy (**t, v**) images of the mediastinum in mice after lymphangiography. Blood was barely detectable in thoracic ducts (arrowheads) of control or *Flcn*$^{i\Delta EC}$ mice. Scale bars: 1 mm (**m, s–v**); 200 µm (**i, j**); 50 µm (**a–h, k, l, o–r**) ; 5 µm (**e–h**) .

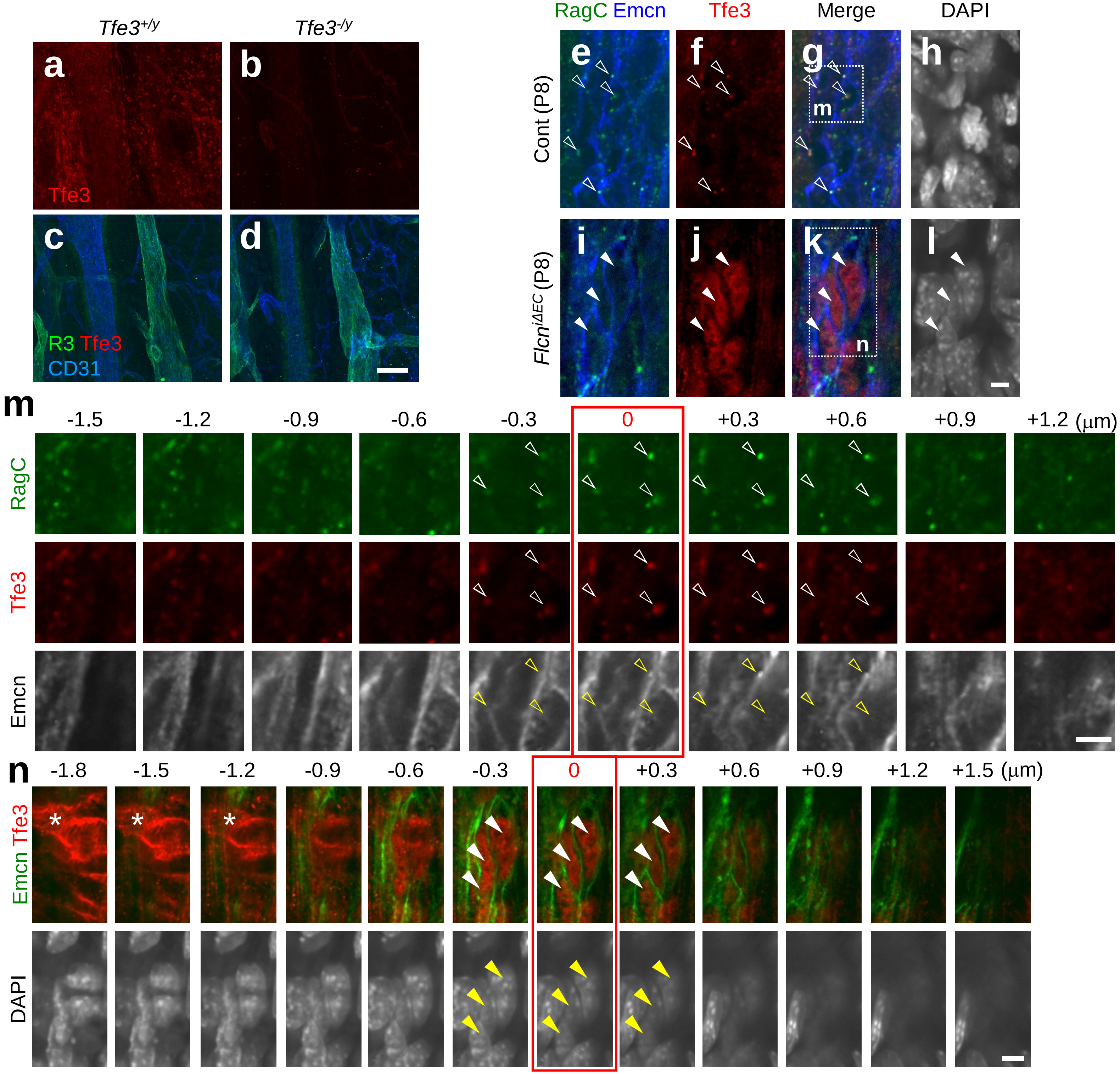

Supplementary Figure 7

Supplementary Figure 7. Expression patterns of Tfe3 in tails.

(**a–d**) Immunohistochemical analysis of whole-mount tails at P8. Tfe3 immunoreactivity, including strong stromal staining seen in the wild-type tail, is not detected in *Tfe3* knockout mice. (**e–n**) Tail whole-mounts at P8. Tfe3 proteins are co-localized with RagC (open arrowheads) in control mice, and translocate into nuclei (closed arrowheads) in $\mathit{Flcn}^{i\Delta EC}$ mice. Panels **m** and **n** indicate images for z-stack slices at 0.3 µm intervals above and below the single slices shown in panels **g** and **k**. Asterisks indicate Tfe3 expression in perivascular (non-endothelial) cells. Scale bars: 50 µm (**a–d**); 5 µm (**e–n**).

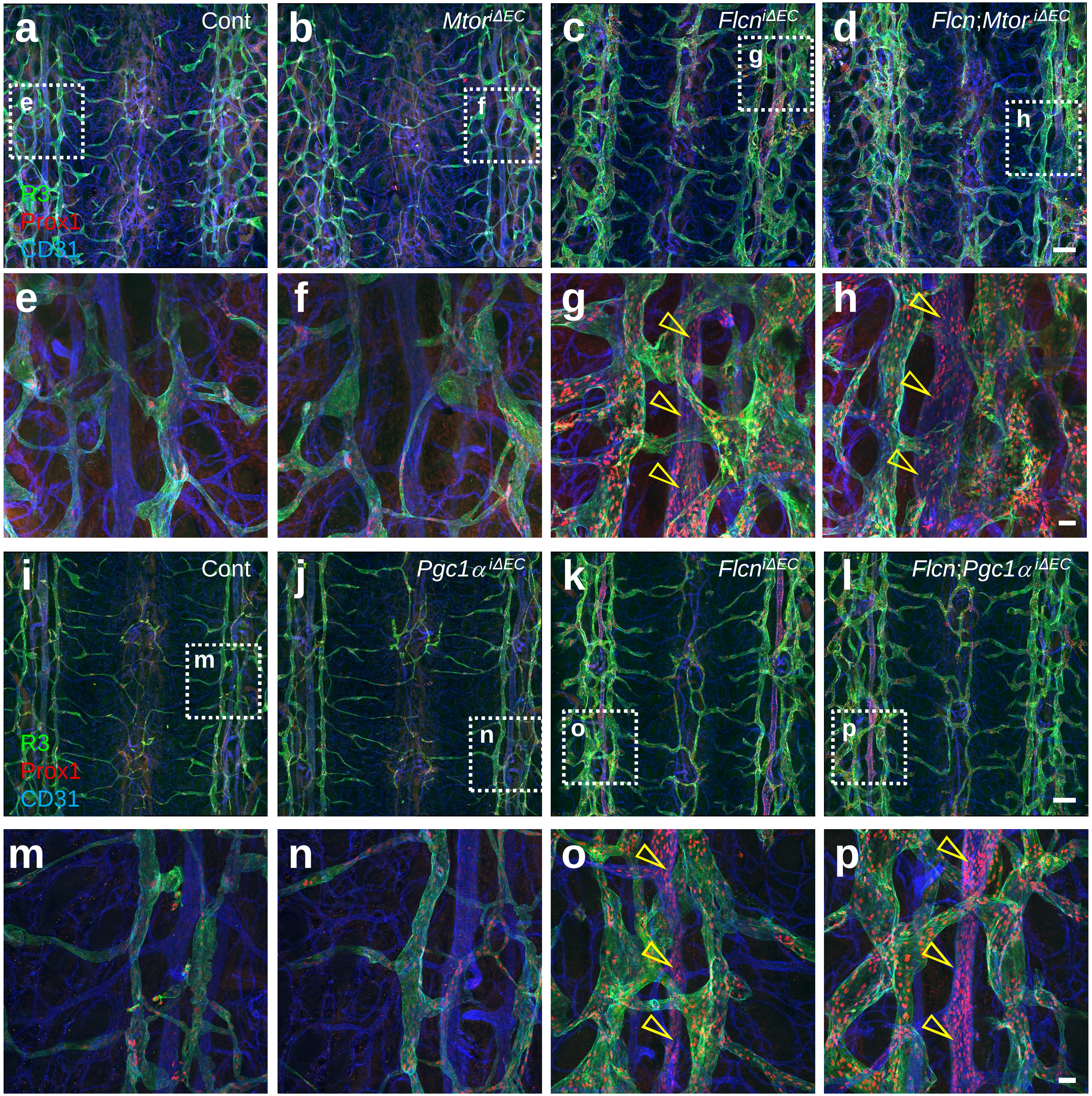

Supplementary Figure 8

Supplementary Figure 8. PGC1α and mTOR are not relevant to phenotypes of Flcn$^{i\Delta EC}$ mice.

(**a–h**) Immunohistochemical analysis of tail whole-mounts at P8. The phenotypes of *Flcn*$^{i\Delta EC}$ mice, including ectopic Prox1 expression in veins (open arrowheads), are not abolished by deletion of *Mtor*. (**i–p**) Immunohistochemical analysis of tail whole-mounts at P8. The phenotypes of *Flcn*$^{i\Delta EC}$ mice, including ectopic Prox1 expression in veins (open arrowheads), are not abolished by deletion of *Pgc1*α. Scale bars: 200 µm (**a–d**, **i–l**); 50 µm (**e–h**, **m–p**).
