# Supplementary material for: Blood and lymphatic systems are segregated by the FLCN tumor suppressor
Source: Nat Commun. 2020 Dec 9;11:6314. doi: 10.1038/s41467-020-20156-6 (PMC7725783; doi:10.1038/s41467-020-20156-6)
Supplement: Supplementary file 3 — Description of Additional Supplementary Files [file 41467_2020_20156_MOESM3_ESM.pdf]

Description of Additional Supplementary Files

File Name: Supplementary Data 1
Description: Genes upregulated in LEC-biased VECs compared with venous endothelial cells (VEC).

File Name: Supplementary Data 2
Description: Genes upregulated in LEC-biased VECs compared with lymphatic endothelial cells (LEC1).

File Name: Supplementary Data 3
Description: Primer list.
