# Supplementary material for: Blood and lymphatic systems are segregated by the FLCN tumor suppressor
Source: Nat Commun. 2020 Dec 9;11:6314. doi: 10.1038/s41467-020-20156-6 (PMC7725783; doi:10.1038/s41467-020-20156-6)

Supplementary Figure-Uncropped scans with size marker indications

Fig. 5l

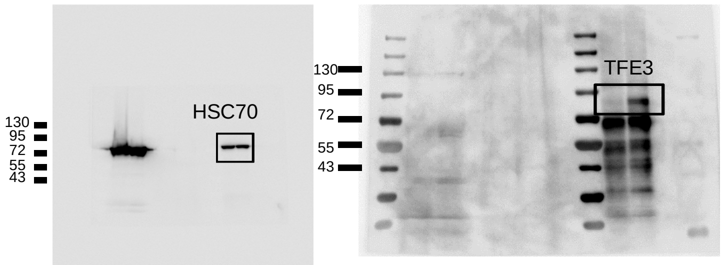

Fig. 5x

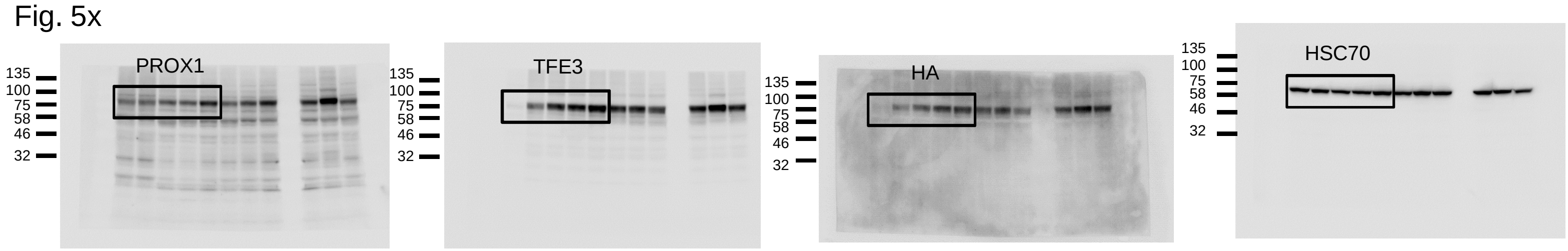

Fig. 6a

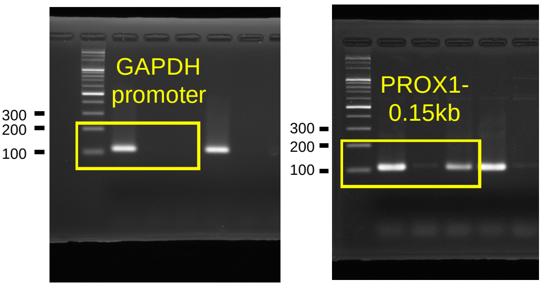

Fig. 6c

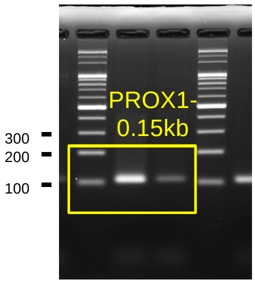

Fig. 6e

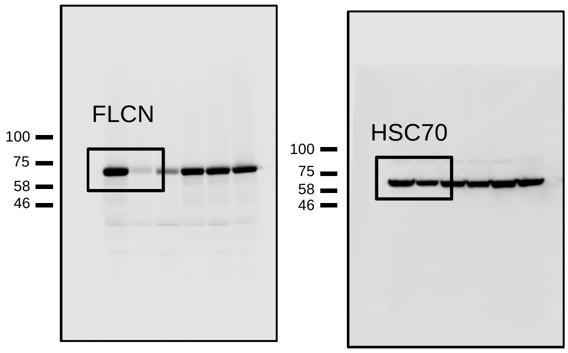

Fig. 6g

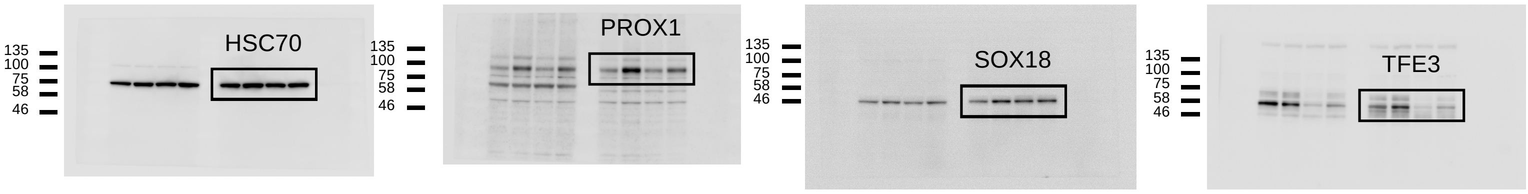

Fig. 7h

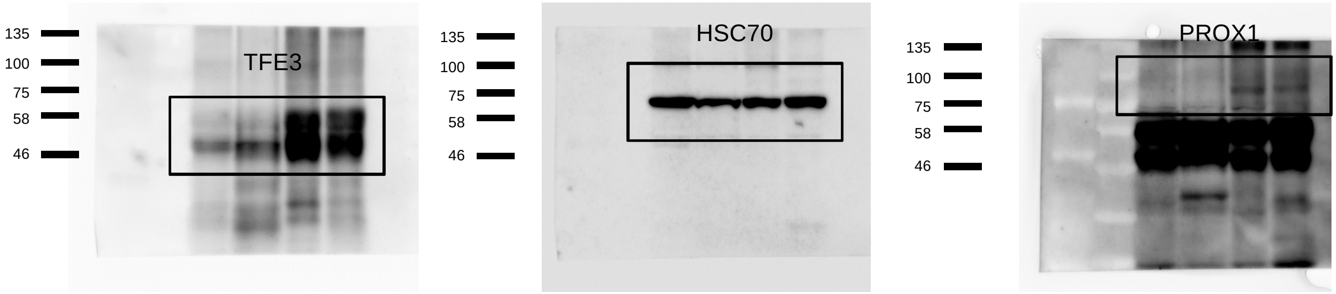

Supple Fig. 2l

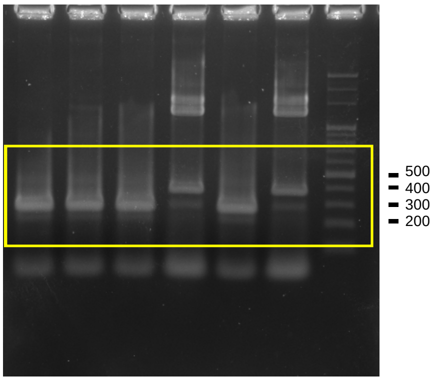

Supplement: Supplementary file 8 — Source Data [file 41467_2020_20156_MOESM8_ESM.zip › Tai-Nagara et al Uncropped gel images.pdf]
